# Spanish and cross-cultural validation of the mind excessively wandering scale

**DOI:** 10.3389/fpsyg.2023.1181294

**Published:** 2023-07-11

**Authors:** Alfonso Morillas-Romero, Alejandro De la Torre-Luque, Florence D. Mowlem, Philip Asherson

**Affiliations:** ^1^Apploading S.L., Palma, Spain; ^2^Department of Psychology of the University of the Balearic Islands, Palma, Spain; ^3^Department of Legal Medicine, Psychiatry and Pathology Department, Complutense University of Madrid, Madrid, Spain; ^4^King’s College London, MRC Social, Genetic and Developmental Psychiatry Centre, Institute of Psychiatry, Psychology and Neuroscience, London, United Kingdom

**Keywords:** mind wandering, task-unrelated thoughts, MEWS, attention, daydreaming

## Abstract

**Introduction:**

Over the last decade, excessive spontaneous mind wandering (MW) has been consistently associated with emotional disorders. The main aims of the present study were (1) to re-examine the factor structure of the Mind Excessively Wandering Scale (MEWS); (2) to validate the Spanish version of the MEWS; and (3) to conduct a cross-cultural validation of the MEWS in Spanish and UK samples.

**Methods:**

A forward/backward translation to Spanish was conducted. Data of 391 Spanish and 713 British non-clinical individuals were analysed.

**Results:**

A revised 10-item version of the MEWS (MEWS-v2.0) demonstrated to be a valid instrument to assess MW. A 2-correlated factor structure properly captured the MEWS-v2.0 variance, accounting for two specific but interrelated dimensions (*Uncontrolled thoughts* and *Mental Overactivity*).

**Discussion:**

The Spanish MEWS-v2.0 showed adequate internal consistency and construct validity, as well as appropriate convergent/divergent validity. Cross-cultural analyses showed that MEWS-v2.0 captured the same construct in both UK and Spanish samples. In conclusion, both Spanish and English MEWS-v2.0 demonstrated to be reliable measures to capture spontaneous MW phenomenon in non-clinical adult populations.

## 1. Introduction

Mind wandering (MW) can be defined as periods of time when attention and the contents of thoughts shift away from external sources and/or ongoing tasks to unrelated internal thoughts or feelings (Smallwood and Schooler, 2015). Over the last decade and following a seminal review by Smallwood and Schooler (2006), the study of MW has become a hot topic in cognitive and affective psychology research (Callard et al., 2013; Christoff et al., 2016; Hobbiss et al., 2019).

Seli et al. (2015) and Carriere et al. (2013) suggested a distinction between intentional/deliberate vs. unintentional/spontaneous MW. Excessive spontaneous MW has been related to several mental health conditions including high levels of neuroticism and anxiety (Christoff et al., 2016; Robison et al., 2017), attention-deficit/hyperactivity disorder (ADHD), and borderline personality disorder (BPD; Mowlem et al., 2016, 2019; Franklin et al., 2017; Moukhtarian et al., 2020); in addition to lower levels of daily happiness (Killingsworth and Gilbert, 2010; Hobbiss et al., 2019), reduced attention, greater interference in performance on executive-function tasks (Smallwood et al., 2004; Mrazek et al., 2012; Mooneyham and Schooler, 2013), reduced dispositional mindfulness (Deng et al., 2014; Marchetti et al., 2016), and increased depressive symptoms (Stawarczyk et al., 2013; Marchetti et al., 2014; Ottaviani et al., 2015). In contrast, potentially linked to deliberate forms of MW, several studies have postulated an adaptive role of MW associated with greater creative problem-solving (Baird et al., 2012; Yamaoka and Yukawa, 2020), adaptive future planning, and better management of personal goals (Baird et al., 2011; Mooneyham and Schooler, 2013; Smallwood and Schooler, 2015).

Several self-reported rating scales have been developed that assess MW. Among these, the Mind Excessively Wandering Scale (MEWS) (Mowlem et al., 2016), is one of the most representative and well-supported by research over the last years. The MEWS development was based on ADHD patient reports of MW and captures both lack of control over MW and difficulty focusing on one thought at a time, thought to be related to spontaneous MW (Mowlem et al., 2019). In this sense, Carriere et al. (2013) developed two separate subscales to distinguish between deliberate and spontaneous MW as two well-differentiated constructs, posteriorly supported by the works of Seli et al. (2015). Related to this, the MEWS showed a strong significant correlation with spontaneous MW but not deliberate MW (Mowlem et al., 2019) suggesting that the MEWS may be considered a specific measure of spontaneous MW. The original MEWS scale consists of 15 items to be scored on a 4-point Likert-type scale (0 = *not at all* or *rarely*, 1 = *some of the time*, 2 = *most of the time*, 3 = *nearly all of the time* or *constantly*), and has shown to be a reliable and valid instrument, demonstrating measurement invariance across sex, age and ADHD diagnostic status (Mowlem et al., 2019).

Following the original authors report, psychometric analysis in a subsequent study showed the MEWS to have a unidimensional structure with good internal consistency and three out of four fit indices suggesting acceptable model fit (Mowlem et al., 2019); specifically, the MEWS showed adequate fit based on the comparative fit index (CFI = 0.97), Tucker-Lewis Index (TLI = 0.99) and standardized root mean square residual (SRMR = 0.06). However, the root mean squared of the residuals (RMSEA) index was 0.13, with some authors suggesting that RMSEA >0.1 0 is indicative of poor fit (Browne and Cudeck, 1993; MacCallum et al., 1996) and be related to inflated type II error rate (Hu and Bentler, 1999). In other words, the higher the RMSEA, the higher the probability of erroneously rejecting a more complex structure of covariances when explaining a construct. In this sense, Nakovics et al. (2020) identified a 2-factorial scale structure solution when analyzing the psychometric properties of the German version of the MEWS. However, the authors argued that these two factors, namely “difficulties controlling own thoughts and focusing” (Factor 1) and “thought fluctuation” (Factor 2), were closely related and interdependent but not distinct facets of MW (Nakovics et al., 2020). Therefore, further research is needed to compare the unidimensional structure of the MEWS and other competing more complex models with large samples.

While the MEWS has been recently adapted and validated in several languages including German (Nakovics et al., 2020) and Portuguese (Figueiredo et al., 2018), a Spanish version is not yet available. Further, to the best of our knowledge, no well-validated self-reported instruments to assess MW are available in the Spanish language for adult populations. Only a Spanish version of the Mind Wandering Questionnaire (MWQ, Mrazek et al., 2013) has been validated in an adolescent sample, replicating the original MWQ factorial structure, but not in adults (Salavera et al., 2017).

The first aim of the present study was to examine the factor structure of the 12-item MEWS using an approach that considered the existence of a potential complex factor structure (hierarchical patterns of variance–covariance) and adapt the scale if required. The second aim was to develop a Spanish version of the MEWS through a backward/forward translation process for use in a Spanish non-clinical adult population to assess the factor structure of the adapted scale in an independent sample. Thirdly, to conduct a cross-sample validation of the Spanish dataset with the UK dataset, to explore the comparability of their construct validity (measurement invariance) in samples from two different cultural backgrounds, and using the different translations of the MEWS. Finally, to investigate the convergent and divergent validity of the Spanish MEWS by exploring its relationships with well-known theoretical constructs. Based on the existing literature, a positive relationship between MW and negative affect (Robison et al., 2017), rumination (Christoff et al., 2016), and anxious and depressive symptomatology was hypothesized (Killingsworth and Gilbert, 2010; Marchetti et al., 2014). In contrast, a negative relationship was expected between MW, and both self-reported attentional control (Mooneyham and Schooler, 2013) and dispositional mindfulness capabilities (Mrazek et al., 2012).

## 2. Materials and methods

### 2.1. Participants

We used two samples from Spain and the United Kingdom. Both samples comprised adults, who voluntarily agreed to participate and signed an informed consent form. Individuals with diagnosed mental disorders were excluded, as well as those with active psychiatric treatment.

The United Kingdom sample consisted of a subsample (*n* = 1,100) extracted from the Mowlem et al. (2019) study database, who had not reported a diagnosis of ADHD (*M* = 33.9 years; *SD* = 13.5; range 18–83 years; 74.2% female). The Spanish sample included 391 students and staff members from the Universitat de les Illes Balears, Universidad Complutense de Madrid, and Universidad de Granada, as well as others in the general population (*M* = 26.9 years; *SD* = 11.9; range 18–70 years; 78% female). Participants were recruited via mailing, electronic, and poster advertisements, as well as through online informative talks, and were not selected on any psychological or sociodemographic characteristics.

The Spanish sample was equivalent to the UK sample in terms of sex proportion, χ^2^ (1) = 2.33, *p* = 0.12, Cramer’s *V* = 0.03. Regarding age, participants from the Spanish sample were younger than those from the UK sample used, *F* (1, 1410) = 82.84, *p* < 0.01, η^2^_partial_ = 0.055. However, age differences did not reach the level of statistical meaningfulness (i.e., medium effect size) to prevent type-I error, in large sample size studies (Lin et al., 2013).

### 2.2. Materials and procedure

Recruitment of the Spanish sample started in the autumn of 2019, being completed half year later. Spanish versions of self-reported measures were collected through online forms. Participants signed an electronic consent form before completing the self-report measures. The Balearic Islands Research Ethics Committee (IB4093/20PI) approved all procedures. Acquisition of self-reported data from the UK used a similar approach (Mowlem et al., 2019).

#### 2.2.1. Translation and adaptation of the Spanish version of the MEWS

Prof. Philip Asherson and Dr. Florence Mowlem provided permission for the translation and adaptation of the MEWS scale (Mowlem et al., 2016). A sequential forward-backward translation approach was followed to adapt the MEWS into Spanish. First, each of the 12 original English items was independently translated into Spanish by two bilingual researchers. Both researchers were familiar with cognitive-related research and clinical practice. Secondly, the proposed translations were discussed, and a consensus was reached prior to the backward translation. Spanish-adapted items were then sent to a native Spanish-English bilingual clinical psychologist to backward translate them again to English. In the third step, two independent researchers and the same bilingual native psychologist compared the item translations and carried out needed variations to ensure a proper content translation (inter-judge content review and correspondence analysis). As a result, the first version of the adapted questionnaire was tested in a pilot sample (*n* = 25) to detect potential difficulties in item comprehension (see Table 1 for final items). No specific difficulties were found in either the items or the instrument overall. After this forward-backward translation, the whole sample was recruited for complete validation analyses.

**Table 1:** Spanish items of the MEWS.

1	Tengo dificultad para controlar mis pensamientos
2	Me resulta difícil apagar/desconectar mis pensamientos
3	Tengo dos o más pensamientos diferentes ocurriendo a la vez
4	Mis pensamientos están desorganizados y fuera de control
5	Mis pensamientos están muy activos todo el tiempo
6	Experimento actividad mental incesante
7	Me resulta difícil pensar en una cosa sin que otro pensamiento entre en mi mente
8	Encuentro que mis pensamientos me distraen y me impiden concentrarme en lo que estoy haciendo
9	Tengo dificultad para reducir la velocidad de mis pensamientos y concentrarme sólo en una cosa a la vez
10	Me resulta difícil pensar con claridad, como si mi mente estuviera nublada
11	Me encuentro revoloteando de un lado a otro entre diferentes pensamientos
12	Sólo puedo enfocar mis pensamientos en una cosa a la vez realizando un esfuerzo considerable

#### 2.2.2. Self-reported negative affect

##### 2.2.2.1. Negative affect

The negative affect subscale of the Positive and Negative Affect Schedule (PANAS, Watson et al., 1988) was used. This subscale consists of 10 statements describing different negative feelings and emotional states rated on a 5-point Likert-type scale (1 = *Not at all*; 5 = *Very much*); measuring the extent to which each statement applies to the person’s global tendencies. Cronbach’s alpha for the Spanish sample was *α* = 0.881.

#### 2.2.3. Self-reported negative emotion regulation strategies

##### 2.2.3.1. Brooding rumination

The shortened Ruminative Response Scale (RRS; Treynor et al., 2003) was used. The scale is composed of 10 items and divided into two subscales: brooding and reflection. Each subscale consists of five items rated on a 4-point Likert-type scale (1 = *Totally disagree*; 4 = *Totally agree*) according to the frequency in which ruminative responses are presented when experiencing a dysphoric mood. Cronbach’s alpha for the Spanish sample was *α* = 0.784.

#### 2.2.4. Self-reported attentional and mindfulness capabilities

##### 2.2.4.1. Attentional control

The Attentional Control Scale (ACS; Derryberry and Reed, 2002) was used. The scale comprises 20 items rated on a 4-point Likert-type scale (1 = *Almost never*; 4 = *Always*) measuring the ability to voluntarily manage attention. The scale can be divided into two subscales: focusing and shifting. Following Ólafsson et al. (2011), item 9 was excluded from the overall score. Cronbach’s alpha for the Spanish sample was *α* = 0.842 for the total scale; and *α* = 0.814 and *α* = 0.729 for focusing and shifting subscales, respectively.

##### 2.2.4.2. Mindfulness

The Spanish version of the Five Facets of Mindfulness Questionnaire (FFMQ; Baer et al., 2006) was used to evaluate self-reported trait mindfulness (Cebolla et al., 2012). It consists of 39 items divided into five subscales assessing different aspects of mindfulness: Observing, Describing, Acting with awareness, Non-judging of inner experience, and Non-reactivity to inner experiences. Items are rated on a Likert scale ranging from 1 (*Never or very rarely true*) to 5 (*Very often or always true*) including some items with reversed scores. Cronbach’s alpha for the Spanish sample was *α* = 0.754 for Observing, *α* = 0.895 for Describing, *α* = 0.862 for Acting with awareness, *α* = 0.879 for Non-judging, and *α* = 0.781 for Non-reactivity.

#### 2.2.5. Depressive and anxiety symptomatology

##### 2.2.5.1. Depressive symptomatology

The Patient Health Questionnaire-9 (PHQ-9) was used. PHQ-9 is a short instrument designed to screen for depression in primary care and other medical settings (Kroenke et al., 2001). Each item is scored from 0 (not at all) to 3 (nearly every day) assessing the concurrent presence of depressive symptomatology. Cronbach’s alpha for the Spanish sample was *α* = 0.884.

##### 2.2.5.2. Anxious symptomatology

The General Anxiety Disorder (GAD-7) instrument (Spitzer et al., 2006) was used to evaluate concurrent anxiety symptoms. The scale has been widely used in clinical practice and research and is composed of 7 items rated from 0 (not at all) to 3 (nearly every day) assessing the concurrent presence of depressive symptomatology. Cronbach’s alpha for the Spanish sample was *α* = 0.886.

### 2.3. Statistical analyses

All the analyses were conducted using the R software (psych, lavaan, and corrplot packages) and SPSS 21.0.0.

#### 2.3.1. Factor structure of the MEWS in the UK dataset

Bearing in mind that the MEWS structure poorly fitted to data, as shown by the elevated close fit testing index in a previous study (RMSEA = 0.015; see Mowlem et al., 2019), model misspecification should not be discarded (Fan and Shivo, 2007; Heene et al., 2012; Savalei, 2012). To deal with covariance structure model misspecification, hierarchical exploratory factor analysis was used. The hierarchical factor analysis allows for detecting complex structures covering common variance entirely explained by subordinate factors (Markon, 2019). This analysis involves transforming an oblique factor solution into an orthogonal solution, ‘preserving the desired interpretation characteristics of the oblique solution, but also discloses the hierarchical structuring of the variables’ (Schmid and Leiman, 1957, p. 53). The hierarchical exploratory factor analyses were then conducted to explore a more complex (hierarchical) structure of the English version of the revised 12-item MEWS (Mowlem et al., 2016) using a UK sample. This may help underlying first-order factors to be visualized.

Confirmatory factor analysis (CFA) was subsequently conducted to compare the fit of factor structures derived from the hierarchical analysis, the 1-factor structure demonstrated in previous studies (Mowlem et al., 2016; Nakovics et al., 2020) and other hierarchical solutions using the UK sample. CFA estimates were obtained using diagonally weighted least square algorithms due to the ordinal response scale of items and data distribution skewness (DiStefano and Morgan, 2014; Li, 2016). Standard errors of estimated parameters were calculated by bootstrapping. The following fit indices were used to assess the goodness of fit (Hu and Bentler, 1999): the χ^2^ test (a non-significant χ^2^ is indicative of perfect fit), the root mean squared of the residuals (RMSEA <0.080 indicates satisfactory fit; RMSEA >0.080 indicates poor fit), the Comparative Fit Index (CFI) and the Tucker-Lewis Index (TLI) (for both indexes scores above 0.95 indicate satisfactory fit) (Hu and Bentler, 1999), and finally, the standardized root mean square residual (SRMR) (scores >0.080 depicting poor fit data).

The hierarchical exploratory factor analysis was conducted on a random subsample comprising data from 30% of the UK sample, following the cross-validation tradition (Knafl and Grey, 2007). Data from the remaining participants were used for CFA.

#### 2.3.2. Validation of the Spanish version of the MEWS

Following translation, CFA on the Spanish translation of the MEWS in the Spanish sample was conducted to test the fit of the optimal factor structure derived from the re-analysis of the MEWS in the UK sample. We then conducted the cross-cultural comparison of factor structure under the measurement invariance (MI) approach (Meredith and Teresi, 2006), which investigates whether the MEWS behaves the same way across the Spanish and UK datasets. According to MI, the fit of models with increasing parameter restrictions is compared: a configural solution (i.e., with the same structural pattern of relationships across samples), metric invariance solution (i.e., constraints on item loadings), and scalar invariance (adding constraints on item thresholds). The incremental CFI (ΔCFI) was used to evaluate measurement invariance. A ΔRMSEA ≥0.015 (in absolute value) and ΔCFI ≥0.010 would reflect significant differences between nested models (Cheung and Rensvold, 2002; Chen, 2007).

To further evaluate the validity of the Spanish MEWS in the Spanish sample, associations between Spanish MEWS scores and PANAS, ACS, RRS, FFMQ, PHQ, and GAD questionnaires were tested as convergent and divergent validity indices using Pearson’s correlation coefficients.

## 3. Results

### 3.1. Descriptive statistics of items response

Descriptive statistics for both Spanish and UK versions of the MEWS items, as well as mean comparisons among them are provided in Table 2.

**Table 2:** Descriptive statistics of item responses and mean comparisons between UK and Spanish samples.

	English MEWS (*n* = 840)			Spanish MEWS (*n* = 391)			
Item	*m*	*sd*	Communality	*m*	*sd*	Communality	*t*
1	2.21	0.89	0.56	2.14	0.81	0.47	−1.29
2	2.66	0.91	0.51	2.58	0.85	0.50	−1.43
3	2.42	0.93	0.45	2.34	0.80	0.32	−1.43
4	2.09	0.93	0.64	1.83	0.79	0.58	−4.67
5	2.51	0.93	0.47	2.84	0.75	0.31	6.02**
6	2.36	0.98	0.52	2.51	0.88	0.36	2.52*
7	2.45	0.88	0.64	2.19	0.78	0.50	−4.88
8	2.24	0.85	0.61	2.21	0.76	0.54	−0.58
9	2.1	0.9	0.72	2.14	0.83	0.57	0.72
10	1.85	0.88	0.53	1.73	0.75	0.46	−2.28
11	2.36	0.89	0.65	2.15	0.81	0.55	−3.86
12	1.98	0.96	0.53	1.89	0.86	0.34	−1.54

*t* = Mean scores comparisons between UK and Spanish samples for each MEWS item; *p < 0.05; **p < 0.01.

No significant differences were found between mean scores in the two versions except for Item 5 and Item 6, although effect sizes were small to moderate (*d_Cohen_* = 0.38; IC95% = 0.25 to 0.50 and *d_Cohen_* = 0.16; IC95% = 0.03 to 0.28, respectively).

### 3.2. Factor structure of the UK sample

As abovementioned, we split our sample into the exploratory factor analysis subsample (*n* = 370) and the confirmatory factor analysis subsample (*n* = 840). The hierarchical factor analysis conducted in the UK exploratory factor analysis sample yielded a 3-factor structure derived from the original 12-item MEWS, explaining 54% of the variance. The standardized loadings derived from this factor structure model under the oblimin rotation are displayed in Table 3.

**Table 3:** Standardized loadings derived from the hierarchical factor analysis on the English MEWS.

Item	F1	F2	F3
1	0.12		**0.81**
2		0.4	**0.54**
3	0.27	**0.48**	
4	**0.59**	0.15	0.12
5		**0.82**	
6		**0.78**	
7	**0.51**	0.24	0.14
8	**0.78**		
9	**0.75**	0.11	
10	**0.83**	−0.11	
11	**0.72**	0.14	
12	**0.81**		

The 3-factor model explained 54% of MEWS variance.

Saturation with higher loading was considered (in bold).

Based on this analysis, we decided to remove Factor 3 due to its weakness, and slightly low reliability (*α* = 0.79), as it had only two saturated items. This involved removing item 1, and as item loading for Item 2 was higher than 0.30 this was considered saturated on factor 2. Item 3 was also removed due to a lack of theoretical consistency with the remaining items that saturated on factor 1. Based on these changes, a revised version of the MEWS (MEWS-v2.0) was adopted. This was a 10-item scale with a 2-factor structure: with Factor 1 (items 4, 7, 8, 9, 10, 11, 12) reflecting *Uncontrolled Thoughts*; and Factor 2 (Items 2, 5, 6) reflecting *Mental Overactivity*.

**Figure 1:**
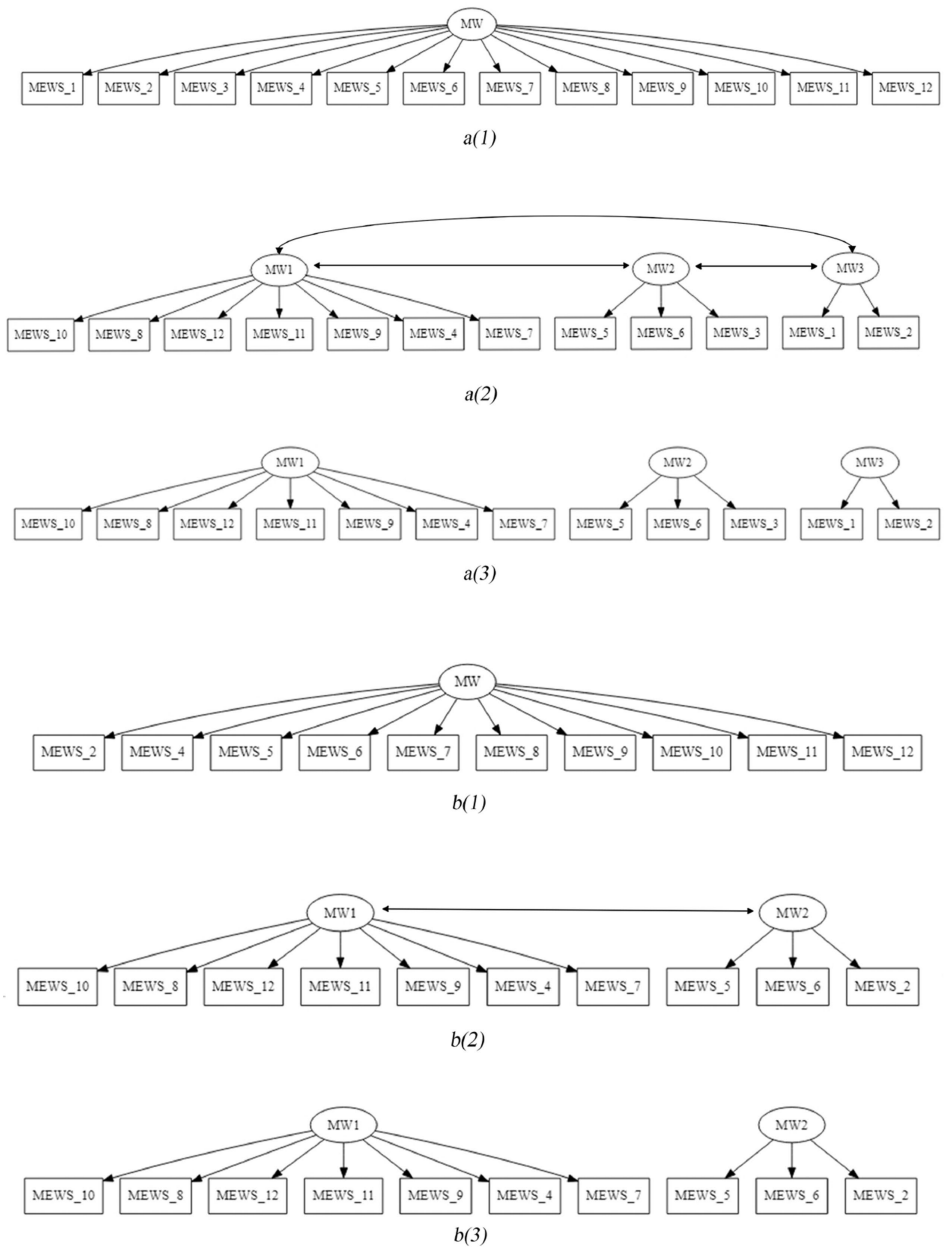
MEWS confirmatory solutions to be tested. *a(1)*, unidimensional 12-item model; *a(2)*, correlated 12-item model; *a(3)*, uncorrelated 12-item model; *b(1)*, unidimensional 10-item model; *b(2)*, correlated 10-item model; *b(3)*, uncorrelated 10-item model.

We conducted the confirmatory factor analysis on the confirmatory factor analysis subsample (*n* = 840). Table 4 displays the fit indexes of the competing confirmatory factor models. Factor models are also depicted in Figure 1.

**Table 4:** Model fit summary of confirmatory factor solutions and reliability index for the English MEWS.

Factor solution	χ^2^ (df)	RMSEA (*CI*_90_)	CFI	TLI	SRMR	Cronbach’s α between factors
12-item version						
1-factor	491.86 (54)	0.107 (0.098, 0.115)	0.992	0.990	0.06	0.94
3-factor (correlated)	197.57 (51)	0.064 (0.054, 0.073)	0.997	0.996	0.04	0.79–0.92
3-factor (uncorrelated)	21882.89 (54)	0.753 (0.745, 0.762)	0.753	0.745	0.44	0.79–0.92
10-item version						
1-factor	401.13 (35)	0.121 (0.111, 0.132)	0.991	0.988	0.07	0.93
**2-factor (correlated)**	**101.87 (34)**	**0.053 (0.041, 0.065)**	**0.998**	**0.998**	**0.04**	0.86–0.92
2-factor (uncorrelated)	10400.19 (35)	0.645 (0.635, 0.655)	0.744	0.671	0.37	0.86–0.92

Model with a better fit in bold.

The 1-factor model came from the original structure of the 12-item MEWS. The 2-factor solution was derived from the hierarchical factor analysis in conjunction with expert guidelines (10 items). The 3-factor solution was derived from the hierarchical factor analysis (12 items).

All the χ^2^-based models were significant, *p* < 0.01.

df, degrees of freedom; RMSEA, Root Mean Square Error of approximation index (scores below 0.080 depict reasonable model fit); CI_90_, confidence interval at 90%; CFI, comparative fit index; TLI, Tucker-Lewis index. Scores of 0.95 or more indicate good model fitting, for TLI and CFI. SRMR, standardized root mean square residual (scores above 0.080 depict poor fit).

Models coming from the hierarchical exploratory factor analysis, using an independent sample.

To ensure manifest variables to be equivalent between the MEWS solutions, we also tested the fit of a 10-item unidimensional model. Therefore, model testing involved comparing two-factor solutions (unifactorial solution vs. the solution derived from the hierarchical exploratory analysis) for the 12-item MEWS (unifactorial solution vs. 3-factor solution, with either correlated or uncorrelated factors), and for the 10-item MEWS (unifactorial solution vs. 2-factor solution, with either correlated or uncorrelated factors). As a result, the correlated factor models (both the 2-factor and 3-factor models with correlated factors) fitted better to data structure, according to fit indexes (i.e., RMSEA <0.080, both CFI and TLI scores >0.95, and, the SRMR <0.080). However, the 3-correlated factor solution yielded a lower Cronbach’s α for one of their factors (*α* < 0.80). Besides, the 2-factor version would better reflect what the instrument measures in theoretical terms. We therefore decided to retain the 2-correlated factor model, with fit indexes: *χ^2^*_(54)_ = 101.87; *p* < 0.01; RMSEA = 0.053, CFI = 0.998, TLI = 0.998, SRMR = 0.04. Factor reliability for the two-correlated factor solution ranged between Cronbach’s *α* = 0.86 to *α* = 0.92.

### 3.3. Cross-cultural validation of the 2-factor solution of the 10-item MEWS (MEWS-v2.0)

To test whether the Spanish MEWS-v2.0 fitted the two-correlated factor solution, a confirmatory factor analysis was conducted. Adequate fit indices were found, χ^2^ (34) = 135.03, *p* < 0.01; RMSEA = 0.067 (*CI*_90_ = 0.058, 0.076), CFI = 0.99, TLI = 0.99, SRMR = 0.06.

Regarding measurement invariance (MI) comparisons to test cross-cultural equivalence of the MEWS structure, fit indices of increasingly restricted solutions are displayed in Table 5. These fit indices reflect an adequate fit of all the MI models. Moreover, item communalities for both UK and Spanish samples were satisfactory, being greater than 0.30 (see Table 2), indicating that the large variance of the item is explained by factors.

**Table 5:** Measurement invariance model comparison to explore cross-cultural effects on MEWS structure.

MI model	χ^2^ (df)	RMSEA (*CI*_90_)	CFI	TLI	SRMR	ΔRMSEA	ΔCFI
Configural	236.91 (68)	0.067 (0.058, 0.077)	0.997	0.996	0.04		
Metric	282.75 (76)	0.070 (0.062, 0.079)	0.996	0.995	0.05	0.003	−0.001
Scalar	351.87 (94)	0.071 (0.063, 0.078)	0.995	0.995	0.04	0.001	−0.001

The configural MI imposes the same structural pattern of relationships between both the English and Spanish MEWS responses. The metric invariance solution imposes constraints on item loadings. The scalar invariance model adds constraints on item thresholds.

All the χ^2^-based models were significant, *p* < 0.01.

MI, Measurement invariance; df, degrees of freedom; RMSEA, Root Mean Square Error of approximation index (scores below 0.080 depict reasonable model fit); CI_90_, confidence interval at 90%; CFI, comparative fit index; TLI, Tucker-Lewis index. Scores of 0.95 or more indicate good model fitting, for TLI and CFI. SRMR, standardized root mean square residual (scores above 0.080 depict poor fit).

Regarding incremental indices, although the incremental CFI might point to a lack of measurement invariance (ΔCFI ≤ −0.01, across model comparisons) this result was not endorsed by ΔRMSEA ≤0.015, suggesting no differences on the MEWS structure parameters (item loadings, intercepts, and residuals) between the English and Spanish datasets.

### 3.4. Convergent and divergent validity in the Spanish sample

Table 6 depicts means and standard deviations for all the self-reported variables included in this study, as well as bivariate correlations between them. The Spanish MEWS-v2.0 (both considering total score and each Factor separately) was positively and significantly associated with negative affect, rumination, and both anxious and depressive symptomatology (stronger correlation value *r* = 0.647). In contrast, the MEWS-v2.0 scores were inversely correlated to attentional control and each of the FFMQ scales but the *Observing* one (stronger correlation value *r* = −0.587). The correlation between MEWS-v2.0 subscales (i.e., Factor 1 and Factor 2) was *r* = 0.599 (*p* < 0.01).

**Table 6:** Descriptive statistics and bivariate correlations between self-reported measures in Spanish sample (*n* = 391).

	*M*	*SD*	1	2	3	4	5	6	7	8	9	10	11	12	13	14	15	16	17
1. MEWS_T	12.06	5.80	1																
2. MEWS_UT	7.13	4.32	0.958**	1															
3. MEWS_MO	4.92	2.06	0.803**	0.599**	1														
4. NA	21.61	1.21	0.620**	0.621**	0.441**	1													
5. RRS_T	23.72	5.62	0.448**	0.431**	0.357**	0.513**	1												
6. RRS_B	11.76	3.53	0.515**	0.519**	0.360**	0.607**	0.829**	1											
7. RRS_R	11.95	3.34	0.210**	0.176**	0.221**	0.222**	0.806**	0.337**	1										
8. ACS_T	49.31	8.60	−0.489**	−0.560**	−0.203**	−0.446**	−0.291**	−0.423**	−0.043	1									
9. ACS_F	23.21	5.05	−0.529**	−0.587**	−0.258**	−0.376**	−0.299**	−0.417**	−0.062	0.892**	1								
10. ACS_S	26.10	4.68	−0.328**	−0.394**	−0.095	−0.412**	−0.212**	−0.326**	−0.013	0.874**	0.560**	1							
11. PHQ-9	8.85	6.10	0.647**	0.638**	0.482**	0.717**	0.497**	0.587**	0.216**	−0.449**	−0.421**	−0.370**	1						
12. GAD-7	8.07	4.92	0.597**	0.587**	0.448**	0.737**	0.483**	0.562**	0.219**	−0.437*	−0.403**	−0.368**	0.764**	1					
13. FFMQ_O	26.60	5.52	0.039	0.012	0.084	−0.020	0.208**	0.070	0.276**	0.103*	0.061	0.123*	0.043	0.055	1				
14. FFMQ_D	25.55	6.85	−0.296**	−0.338**	−0.124**	−0.292**	−0.095	−0.288**	0.145**	0.310**	0.274**	0.274*	−0.331**	−0.260**	0.285**	1			
15. FFMQ_A	23.30	5.96	−0.505**	−0.538**	−0.292**	−0.349**	−0.242**	−0.308**	−0.081	0.489**	0.506**	0.353**	−0.371**	−0.321**	0.023	0.272**	1		
16. FFMQ_NJ	23.84	7.02	−0.398**	−0.409**	−0.263**	−0.464**	−0.273**	−0.406**	−0.030	0.257**	0.236**	0.219**	−0.435**	−0.416**	0.044	0.378**	0.359**	1	
17. FFMQ_NR	20.41	4.50	−0.452**	−0.459**	−0.307**	−0.465**	−0.230**	−0.365**	−0.001	0.324**	0.279**	0.294**	−0.416**	−0.434**	0.276**	0.297**	0.175**	3.91**	1

M, mean; SD, Standard Deviation; MEWS_T, MEWS Total Score; MEWS_UT, MEWS Uncontrolled Thoughts Subscale; MEWS_MO, MEWS Mental Overreactivity Subscale; NA, PANAS Negative Affect Subscale; RRS_T, RRS Total Score; RRS_B, RRS Brooding Subscale; RRS_R, RRS Reflection Subscale; ACS_T, Attentional Control Scale Total; ACS_F, ACS Focusing Subscale; ACS_S, ACS Shifting Subscale; FFMQ_O, Five Facets Mindfulness Questionnaire Observing Subscale; FFMQ_D, Five Facets Mindfulness Questionnaire Describing Subscale; FFMQ_A, Five Facets Mindfulness Questionnaire Awarenness Subscale; FFMQ_NJ, Five Facets Mindfulness Questionnaire Non-Judgment Subscale; FFMQ_NR, Five Facets Mindfulness Questionnaire Non-reaction Subscale.

*p < 0.005; **p < 0.000.

## 4. Discussion

The first aim of this study was to re-analyze the English version of the 12-item MEWS (Mowlem et al., 2016) in a UK sample, to explore alternative factor structures of the instrument under hierarchical models. This led to a revised 10-item version of the scale (MEWS-v2.0). The second aim was to translate the MEWS to Spanish and assess the cross-scale validity of the translations by evaluating the factor structure of the adapted MEWS-v2.0. Further validation steps for the MEWS-v2.0 included cross-cultural comparisons of the English and Spanish datasets for measurement invariance; and convergent validity against clinical scales previously associated with MW, in the Spanish sample.

We re-evaluated the factor structure of the original 12-item MEWS in the UK sample using a hierarchical approach, given the inflated RMSEA (RMSEA >0.10) of the unidimensional MEWS (Mowlem et al., 2019). Our results showed that a shorter 10-item version of the MEWS (MEWS-v2.0) with a 2-correlated factor structure, had a better fit to data than the original unidimensional structure or a 3-factor solution on the original 12-item version. More specifically, our results, therefore, suggest the existence of two factors which, given the item content, could fit the descriptive labels of *Uncontrolled Thoughts* (Factor 1) and *Mental Overactivity* (Factor 2). Although the hierarchical EFA also suggested a 3-factor solution, with satisfactory CFA fit indices, we decided to remove the factor 3 due to factor overdetermination issues and factor stability (MacCallum et al., 1996; Tabachnick and Fidell, 2001). We come from the assumption that a factor with fewer than three items may be distinctive of a weak factor (Costello and Osborne, 2005). Two items were saturated on Factor 3 (the item 1 and 2). The item 2 saturated on both Factor 2 and 3, leading to cross-loading issues. In this regard, cross-loading items (i.e., those with loading at least 0.32 on two or more factors) may affect the accuracy of factor extraction based on eigenvalue methods (Li et al., 2020). We decided the item 2 to be dropped from Factor 3 and retained on Factor 2, as its loading was higher than 0.30 and it theoretically matches with the Factor 2 construct (Mental overactivity). Only one item distinctively saturated on this third factor (item 1). We, therefore, decided to drop item 1 from the scale so as to retain robust factors from the MEWS.

The MEWS-v2.0 may be used both as a unidimensional scale to assess the global tendency to engage in excessive spontaneous MW, as well as divided in two interdependent subscales of *Uncontrolled thoughts* and *Mental Overactivity*. Although highly correlated, both factors (*Uncontrolled thoughts* and *Mental Overactivity*) appear to reflect two theoretically separated constructs. While *Mental Overactivity* items’ content reflects a greater tendency to experience thoughts constantly on the go and flitting from one topic to another, *Uncontrolled Thoughts* reflect a person’s difficulty in *voluntarily regulating* these unfocused thoughts. Therefore, they appear to relate closely to concepts of attentional and cognitive control. This proposal aligns with studies suggesting that reduced attentional control may be a shared mechanism between MW and external distraction (Unsworth et al., 2012). In relation to the Christoff et al. (2016) model, it is tempting to speculate that participants with a greater tendency to MW who also exhibit greater attentional control capabilities, would show more controlled forms of spontaneous thought. For example, off-task thoughts might be more deliberate and potentially more creative, reflecting a more deliberate form of MW. Further studies would benefit from explicitly exploring this hypothesis by comparing the MEWS-v2.0 subscales derived from the 2-factors structure with other scales addressing different aspects of MW; such as deliberate and spontaneous forms of MW (Mrazek et al., 2013).

Although our data-driven approach generated two factors, both appeared to be highly correlated, which suggests that they are interdependent and reflect different aspects of the same higher global dimension; that is spontaneous MW. This conceptualisation seems to be in line with the results of the factorial structure of the German version of the MEWS (MEWS-G) reported by Nakovics et al. (2020), as their explorative analyses found that both a 2-factor and 3-factor solutions explained greater variance than a unidimensional factor structure. However, they also found those models to have many cross-loadings with many items saturating in different factors at the same time; reflecting an overlap between factors consistent with a single global dimension (Nakovics et al., 2020).

Regarding the new Spanish version of the 10-item MEWS-v2.0, CFA analysis showed a good fit in line with the results derived from the re-analysis of the English MEWS, with the 2-correlated factor structure fitted adequately to data. Values of Cronbach’s α reflected that internal consistency of the two-factor model was high both for the Spanish and UK 10-item versions in their respective samples. Additionally, the cross-cultural analyses showed measurement invariance, suggesting that MEWS-v2.0 is a reliable and valid instrument capturing the same constructs across both UK and Spanish samples; so that no cultural, cross-sample, or scale translation differences were observed. These findings strongly support the validity of the forward-backward translation process and the adaptation of the MEWS to a revised 10-item version.

The Spanish MEWS-v2.0 also demonstrated reliable convergent and divergent validity. Starting with convergent measures, a positive relationship was found between the self-reported tendency to excessive MW and rumination. These associations were significant when considering both total and subscale-divided MEWS-v2.0 scores. This should not be surprising given the substantial overlap between the core characteristics of both constructs. For example, both brooding rumination and spontaneous MW seem to share an unintentional nature. Furthermore, they are both associated with executive impairments since individuals with a greater tendency to MW (and/or ruminate) would exhibit greater difficulties in disengaging attentional resources from irrelevant-task stimuli, and ultimately controlling thoughts. Our results are in line with this conception, showing both MEWS-v2.0 subscales to be negatively related to the self-reported capability to voluntarily manage attentional resources.

Christoff et al. (2016) recently described that MW can be characterized by a huge variability in the content of thought, whereas brooding rumination would tend to remain fixed/restricted on a single and negatively valenced topic. Thus, they consider rumination to be a constrained form of MW in terms of content (Christoff et al., 2016). However, previous studies also reported independent electrophysiological indices (heart rate variability), associated with each of them (Ottaviani et al., 2015). Further complimentary studies would benefit by combining cardiac indices with experience-sampling methods when analyzing MW, considering both contents of thought and contextual variables.

A significant positive relationship between MW and anxious and depressive symptomatology, as well as with negative affect was found in the Spanish sample. This is in line with previous literature linking excessive MW to neuroticism (Robison et al., 2017) and depressive symptoms (Killingsworth and Gilbert, 2010; Andrews-Hanna et al., 2013; Hoffmann et al., 2016). However, the mechanism of the MW-Negative mood pathway remains unclear and needs further investigation, since the direction of any causal relationship is still not well understood (Smallwood and O’Connor, 2011; Stawarczyk et al., 2013).

Regarding divergent validity, as expected, a significant negative association was found between attentional control and MW, with those reporting higher MW scores, reporting lower attentional control capabilities. Although, impaired task performance for measures of attentional skills has previously been reported to be related to high levels of MW (Mooneyham and Schooler, 2013; Stawarczyk et al., 2014), evidence for this association using self-reported measures of attentional control remained unexplored in adults. Assuming an equivalence between performance-based and self-reported measures of attentional control [although this has been questioned (Tortella-Feliu et al., 2014)], a negative association between self-reported attentional control and MW was expected. This makes even more sense considering that reduced self-reported attentional control has been previously related to an increased tendency to ruminate (Armstrong et al., 2011; Koster et al., 2011; Tortella-Feliu et al., 2014). It may be hypothesized that both MW and rumination share some underlying mechanisms, such as low attentional control, which may be at least partially responsible for their commonalities. Attentional control refers to the ability to voluntarily regulate and manage attentional allocation, including the capability to concentrate and resist distraction, to switch attention between tasks, and to flexibly control thoughts (Derryberry and Reed, 2002). It is therefore plausible that participants with lower attentional control capabilities find it harder to disengage their attention from self-focused task-unrelated thoughts and redirect attentional focus to an ongoing task. In line with this, Forster and Lavie (2009, 2014) advanced the hypothesis that MW could be a manifestation of a more general susceptibility to irrelevant distractions, whether from internal task unrelated thoughts or external distractions.

Finally, it has been proposed that there is an opposite relationship between mindfulness capabilities and MW (Marchetti et al., 2016), since the ability to remain mindful at the moment (i.e., focused on an object or task) appears to be in direct opposition to the tendency for attention to wander away from the task at hand (Mrazek et al., 2012). Consistent with this, our data showed that an increased tendency to MW was significantly and negatively associated with four out of five of the FFMQ subscales (Describing, Awareness, Non-judgment, and Non-reaction). The Observing subscale of the FFMQ appeared not to be associated with either the total MEWS-v2.0 score or any of the two subscales. Considering that this FFMQ subscale reflects the ability to notice or attend to internal and external experiences, such as thoughts, sensations, or emotions (Baer et al., 2006), this was unexpected. However, our results are in line with those reported by Cebolla et al. (2012) who found that the Observing subscale was the only one not showing convergent and divergent validity. It is tempting to speculate that this may be linked with an unawareness MW experience, and further studies would benefit to include awareness-unawareness as key dimensions when studying MW in relation to emotional-related processes (Schooler et al., 2011).

This study has some limitations. First, our main aim was to validate the scale as an instrument to evaluate individual differences in MW in the general adult population, so participants meeting the criteria for mental health disorders were excluded from this study. Furthermore, the female gender constituted the 78% and the 74.2% of the Spanish and UK sample respectively, which may affect the representativeness of the sample. In order to contribute to the generalisability of the results, future studies would benefit from a more gender-equitable sample. In relation to the Spanish version of the MEWS, future studies would benefit from analysis in clinical populations, such as ADHD, to ensure measurement invariance between general and clinical population samples. Secondly, the non-clinical UK sample analyzed in this study was selected from a broader pool of participants under the unique condition of not being diagnosed with ADHD. It may however have been that some of these may have presented with other mental health issues which have not been accounted for. Thirdly, further studies will need to explore the convergent validity of the MEWS-v2.0 and more specifically the new Spanish translation, with other scales addressing different aspects of MW such as deliberate and spontaneous forms of MW.

Several studies have already begun to include experience sampling-based measures of MW and executive/attentional performance-based measures, as a complement of self-reported instruments when exploring emotional-related factors, such as emotional instability (Moukhtarian et al., 2020; Bozhilova et al., 2021). This multi-method approach is of special interest to better capture and understand the MW phenomenon and to contribute to the exploration of its potential relationships with basic attentional impairments. Additionally, results transferability from laboratory settings to ecological environments as related to MW seems to be a controversial issue (Kane et al., 2017; Linz et al., 2021) and further research combining these measures is still needed.

## 5. Conclusion

The 10-item version of both the English and the Spanish MEWS (MEWS-v2.0) were demonstrated to be useful and valid instruments to assess MW in healthy adult populations. Two factors were identified reflecting *Uncontrolled thoughts* (Factor 1) and *Mental Overactivity* (Factor 2). The correlated two-factor structure may optimally capture the MEWS variance, accounting for two specific but interrelated dimensions of a higher dimension of spontaneous MW. The Spanish version of MEWS-v2.0 showed adequate internal consistency levels and construct validity, as well as evidence of convergent and divergent validity in line with hypothesized relationship directions between selected measures. Furthermore, the cross-cultural analyses showed that the Spanish MEWS-v2.0 was a reliable and valid instrument capturing the same construct as the English version of MEWS-v2.0. Given that excessive spontaneous MW has been previously associated with several clinical manifestations such as depression, ADHD, and related factors such as negative affect and executive impairments, it is tempting to hypothesize its role as a transdiagnostic process. However, further research is needed to contribute to our understanding of the functional consequences of MW and explore the potential role of MW as related to vulnerability to affective disorders. Additionally, MW needs to be also considered as a potential clinical treatment target, for which proper assessment tools are critical.

## Data availability statement

The raw data supporting the conclusions of this article will be made available by the authors, without undue reservation.

## Ethics statement

The studies involving human participants were reviewed and approved by the Balearic Islands Research Ethics Committee (IB4093/20PI). The patients/participants provided their written informed consent to participate in this study.

## Author contributions

AM-R: conceptualization, methodology, investigation, formal analysis, and original draft preparation. AT-L: formal analysis, data curation, and original draft preparation. FM and PA: reviewing, editing, and resources. All authors contributed to the article and approved the submitted version.

## Funding

This work was supported by the Spanish Government through the Torres Quevedo Grant Program (PTQ2018-009836) and the Department of Psychology of the University of the Balearic Islands.

## Conflict of interest

AM-R was employed by company Apploading S.L.

The remaining authors declare that the research was conducted in the absence of any commercial or financial relationships that could be construed as a potential conflict of interest.

## Publisher’s note

All claims expressed in this article are solely those of the authors and do not necessarily represent those of their affiliated organizations, or those of the publisher, the editors and the reviewers. Any product that may be evaluated in this article, or claim that may be made by its manufacturer, is not guaranteed or endorsed by the publisher.

## References

[ref1] Andrews-Hanna J. R. Kaiser R. H. Turner A. E. J. Reineberg A. E. Godinez D. Dimidjian S.. (2013). A penny for your thoughts: dimensions of self-generated thought content and relationships with individual differences in emotional wellbeing. Front. Psychol. 4:900. doi: 10.3389/fpsyg.2013.00900 24376427 PMC3843223

[ref2] Armstrong T. Zald D. H. Olatunji B. O. (2011). Attentional control in OCD and GAD: specificity and associations with core cognitive symptoms. Behav. Res. Ther. 49, 756–762. doi: 10.1016/j.brat.2011.08.003, PMID: 21871608 PMC3266065

[ref3] Baer R. A. Smith G. T. Hopkins J. Krietemeyer J. Toney L. (2006). Using self-report assessment methods to explore facets of mindfulness. Assessment 13, 27–45. doi: 10.1177/1073191105283504, PMID: 16443717

[ref4] Baird B. Smallwood J. Mrazek M. D. Kam J. W. Y. Franklin M. S. Schooler J. W. (2012). Inspired by distraction: mind wandering facilitates creative incubation. Psychol. Sci. 23, 1117–1122. doi: 10.1177/0956797612446024 22941876

[ref5] Baird B. Smallwood J. Schooler J. W. (2011). Back to the future: autobiographical planning and the functionality of mind-wandering. Conscious. Cogn. 20, 1604–1611. doi: 10.1016/j.concog.2011.08.007, PMID: 21917482

[ref6] Bozhilova N. Kuntsi J. Rubia K. Michelini G. Asherson P. (2021). Electrophysiological modulation of sensory and attentional processes during mind wandering in attention-deficit/hyperactivity disorder. Neuroimage 29:102547. doi: 10.1016/j.nicl.2020.102547, PMID: 33444949 PMC7808945

[ref7] Browne M. W. Cudeck R. (1993). “Alternative ways of assessing model fit” in Testing structural equation models. eds. Bolle K. A. Long J. S. (Newbury Park, CA: Sage), 136–162.

[ref8] Callard F. Smallwood J. Golchert J. Margulies D. S. (2013). The era of the wandering mind? Twenty-first century research on self-generated mental activity. Front. Psychol. 4:891. doi: 10.3389/fpsyg.2013.00891, PMID: 24391606 PMC3866909

[ref9] Carriere J. S. A. Seli P. Smilek D. (2013). Wandering in both mind and body: individual differences in mind wandering and inattention predict fidgeting. Can. J. Exp. Psychol. 67, 19–31. doi: 10.1037/a0031438, PMID: 23458548

[ref10] Cebolla A. García-Palacios A. Soler J. Guillen V. Baños R. Botella C. (2012). Psychometric properties of the Spanish validation of the five facets of mindfulness questionnaire (FFMQ). Eur. J. Psychiatry 26, 118–126. doi: 10.4321/S0213-61632012000200005

[ref11] Chen F. F. (2007). Sensitivity of goodness of fit indexes to lack of measurement invariance. Struct. Equ. Model. Multidiscip. J. 14, 464–504. doi: 10.1080/10705510701301834

[ref12] Cheung G. W. Rensvold R. B. (2002). Evaluating goodness-of-fit indexes for testing measurement invariance. Struct. Equ. Model. Multidiscip. J. 9, 233–255. doi: 10.1207/S15328007SEM0902_5

[ref13] Christoff K. Irving Z. C. Fox K. C. R. Nathan Spreng R. Andrews-Hanna J. R. (2016). Mind-wandering as spontaneous thought: a dynamic framework. Nat. Rev. Neurosci. 17, 718–731. doi: 10.1038/nrn.2016.113 27654862

[ref14] Costello A. B. Osborne J. (2005). Best practices in exploratory factor analysis: four recommendations for getting the most from your analysis. Pract. Assess. Res. Eval. 10, 1–9. doi: 10.7275/jyj1-4868

[ref15] Deng Y.-Q. Li S. Tang Y.-Y. (2014). The relationship between wandering mind, depression and mindfulness. Mindfulness 5, 124–128. doi: 10.1007/s12671-012-0157-7

[ref16] Derryberry D. Reed M. A. (2002). Anxiety-related attentional biases and their regulation by attentional control. J. Abnorm. Psychol. 111, 225–236. doi: 10.1037/0021-843X.111.2.225, PMID: 12003445

[ref17] DiStefano C. Morgan G. B. (2014). A comparison of diagonal weighted least squares robust estimation techniques for ordinal data. Struct. Equ. Model. Multidiscip. J. 21, 425–438. doi: 10.1080/10705511.2014.915373

[ref18] Fan X. Shivo S. A. (2007). Sensitivity of fit indices to model misspecification and model types. Multivar. Behav. Res. 42, 509–529. doi: 10.1080/00273170701382864

[ref19] Figueiredo T. Erthal P. Fortes D. Asherson P. Mattos P. (2018). Transcultural adaptation to Portuguese of the mind excessively wandering scale (MEWS) for evaluation of thought activity. Trends Psychiatry Psychotherapy 40, 337–341. doi: 10.1590/2237-6089-2017-0117, PMID: 30234888

[ref20] Forster S. Lavie N. (2009). Harnessing the wandering mind: the role of perceptual load. Cognition 111, 345–355. doi: 10.1016/j.cognition.2009.02.006, PMID: 19327760 PMC2706319

[ref21] Forster S. Lavie N. (2014). Distracted by your mind? Individual differences in distractibility predict mind wandering. J. Exp. Psychol. Learn. Mem. Cogn. 40, 251–260. doi: 10.1037/a0034108, PMID: 23957365

[ref22] Franklin M. S. Mrazek M. D. Anderson C. L. Johnston C. Smallwood J. Kingstone A.. (2017). Tracking distraction. J. Atten. Disord. 21, 475–486. doi: 10.1177/1087054714543494 25085650

[ref23] Heene M. Sven Hilbert H. Freudenthaler H. Bühner M. (2012). Sensitivity of SEM fit indexes with respect to violations of uncorrelated errors. Struct. Equ. Model. Multidiscip. J. 19, 36–50. doi: 10.1080/10705511.2012.634710

[ref24] Hobbiss M. H. Fairnie J. Jafari K. Lavie N. (2019). Attention, Mindwandering, and mood. Conscious. Cogn. 72, 1–18. doi: 10.1016/j.concog.2019.04.007, PMID: 31059975

[ref25] Hoffmann F. Banzhaf C. Kanske P. Bermpohl F. Singer T. (2016). Where the depressed mind wanders: self-generated thought patterns as assessed through experience sampling as a state marker of depression. J. Affect. Disord. 198, 127–134. doi: 10.1016/j.jad.2016.03.005, PMID: 27015160

[ref26] Hu L. T. Bentler P. M. (1999). Cutoff criteria for fit indexes in covariance structure analysis: conventional criteria versus new alternatives, structural equation modeling. Struct. Equ. Model. 6, 1–55. doi: 10.1080/10705519909540118

[ref27] Kane M. J. Gross G. M. Chun C. A. Smeekens B. A. Meier M. E. Silvia P. J.. (2017). For whom the mind wanders, and when, varies across laboratory and daily-life settings. Psychol. Sci. 28, 1271–1289. doi: 10.1177/0956797617706086, PMID: 28719760 PMC5591044

[ref28] Killingsworth M. A. Gilbert D. T. (2010). A wandering mind is an unhappy mind. Science 330:932. doi: 10.1126/science.1192439, PMID: 21071660

[ref29] Knafl G. J. Grey M. (2007). Factor analysis model evaluation through likelihood cross-validation. Stat. Methods Med. Res. 16, 77–102. doi: 10.1177/0962280206070649, PMID: 17484294 PMC2984549

[ref30] Koster E. H. W. De Lissnyder E. Derakshan N. De Raedt R. (2011). Understanding depressive rumination from a cognitive science perspective: the impaired disengagement hypothesis. Clin. Psychol. Rev. 31, 138–145. doi: 10.1016/j.cpr.2010.08.005, PMID: 20817334

[ref31] Kroenke K. Spitzer R. L. Williams J. B. (2001). The PHQ-9: validity of a brief depression severity measure. J. Gen. Intern. Med. 16, 606–613. doi: 10.1046/j.1525-1497.2001.016009606.x, PMID: 11556941 PMC1495268

[ref32] Li C. H. (2016). Confirmatory factor analysis with ordinal data: comparing robust maximum likelihood and diagonally weighted least squares. Behav. Res. Methods 48, 936–949. doi: 10.3758/s13428-015-0619-7, PMID: 26174714

[ref33] Li Y. Wen Z. Hau K.-T. Yuan K.-H. Peng Y. (2020). Effects of cross-loadings on determining the number of factors to retain. Struct. Equ. Model. Multidiscip. J. 27, 841–863. doi: 10.1080/10705511.2020.1745075

[ref34] Lin M. Lucas H. C. Jr. Shmueli G. (2013). Research commentary—too big to fail: large samples and the p-value problem. Inform. Syst. Res. 24, 906–917. doi: 10.1287/isre.2013.0480

[ref35] Linz R. Pauly R. Smallwood J. Engert V. (2021). Mind-wandering content differentially translates from lab to daily life and relates to subjective stress experience. Psychol. Res. 85, 649–659. doi: 10.1007/s00426-019-01275-2, PMID: 31832761 PMC7900029

[ref36] MacCallum R. C. Browne M. W. Sugawara H. M. (1996). Power analysis and determination of sample size for covariance structure modeling. Psychol. Methods 1, 130–149. doi: 10.1037/1082-989X.1.2.130

[ref37] Marchetti I. Koster E. H. W. Klinger E. Alloy L. B. (2016). Spontaneous thought and vulnerability to mood disorders: the dark side of the wandering mind. Clin. Psychol. Sci. 4, 835–857. doi: 10.1177/2167702615622383, PMID: 28785510 PMC5544025

[ref38] Marchetti I. Van de Putte E. Koster E. H. W. (2014). Self-generated thoughts and depression: from daydreaming to depressive symptoms. Front. Hum. Neurosci. 8:131. doi: 10.3389/fnhum.2014.00131, PMID: 24672458 PMC3957030

[ref39] Markon K. E. (2019). Bifactor and hierarchical models: specification, inference, and interpretation. Annu. Rev. Clin. Psychol. 15, 51–69. doi: 10.1146/annurev-clinpsy-050718-095522, PMID: 30649927

[ref40] Meredith W. Teresi J. A. (2006). An essay on measurement and factorial invariance. Med. Care 44, S69–S77. doi: 10.1097/01.mlr.0000245438.73837.89, PMID: 17060838

[ref41] Mooneyham B. W. Schooler J. W. (2013). The costs and benefits of mind-wandering: a review. Can. J. Exp. Psychol. 67, 11–18. doi: 10.1037/a0031569 23458547

[ref42] Moukhtarian T. R. Reinhard I. Morillas-Romero A. Ryckaert C. Mowlem F. Bozhilova N.. (2020). Wandering minds in attention-deficit/hyperactivity disorder and borderline personality disorder. Eur. Neuropsychopharmacol. 38, 98–109. doi: 10.1016/j.euroneuro.2020.07.005, PMID: 32703662

[ref43] Mowlem F. D. Agnew-Blais J. Pingault J. B. Asherson P. (2019). Evaluating a scale of excessive mind wandering among males and females with and without attention-deficit/hyperactivity disorder from a population sample. Sci. Rep. 9:3071. doi: 10.1038/s41598-019-39227-w, PMID: 30816143 PMC6395591

[ref44] Mowlem F. D. Skirrow C. Reid P. Maltezos S. Nijjar S. K. Merwood A.. (2016). Validation of the mind excessively wandering scale and the relationship of mind wandering to impairment in adult ADHD. J. Atten. Disord. 23, 624–634. doi: 10.1177/1087054716651927, PMID: 27255536 PMC6429624

[ref45] Mrazek M. D. Phillips D. T. Franklin M. S. Broadway J. M. Schooler J. W. (2013). Young and restless: validation of the mind-wandering questionnaire (MWQ) reveals disruptive impact of mind-wandering for youth. Front. Psychol. 4:560. doi: 10.3389/fpsyg.2013.00560, PMID: 23986739 PMC3753539

[ref46] Mrazek M. D. Smallwood J. Schooler J. W. (2012). Mindfulness and mind-wandering: finding convergence through opposing constructs. Emotion 12, 442–448. doi: 10.1037/a0026678, PMID: 22309719

[ref47] Nakovics H. Benoit D. Asherson P. Luderer M. Alm B. Vollstädt-Klein S.. (2020). Validation of the German version of the mind excessively wandering scale (MEWS-G). Fortschritte Der Neurologie Psychiatrie. 89, 607–616. doi: 10.1055/a-1362-9743, PMID: 33657626

[ref48] Ólafsson R. P. Smári J. Guðmundsdóttir F. Olafsdóttir G. Harðardóttir H. L. Einarsson S. M. (2011). Self reported attentional control with the attentional control scale: factor structure and relationship with symptoms of anxiety and depression. J. Anxiety Disord. 25, 777–782. doi: 10.1016/j.janxdis.2011.03.013, PMID: 21531115

[ref49] Ottaviani C. Shahabi L. Tarvainen M. Cook I. Abrams M. Shapiro D. (2015). Cognitive, behavioral, and autonomic correlates of mind wandering and perseverative cognition in major depression. Front. Neurosci. 9:433. doi: 10.3389/fnins.2014.00433 25601824 PMC4283544

[ref50] Robison M. K. Gath K. I. Unsworth N. (2017). The neurotic wandering mind: an individual differences investigation of neuroticism, mind-wandering, and executive control. Q. J. Exp. Psychol. 70, 649–663. doi: 10.1080/17470218.2016.1145706, PMID: 26821933

[ref51] Salavera C. Urcola-Pardo F. Usán P. Jarie L. (2017). Translation and validation of the mind-wandering test for Spanish adolescents. Psicologia 30. doi: 10.1186/s41155-017-0066-8 PMC6963992 32026091

[ref52] Savalei V. (2012). The relationship between root mean square error of approximation and model misspecification in confirmatory factor analysis models. Educ. Psychol. Meas. 72, 910–932. doi: 10.1177/001316441245256

[ref53] Schmid J. Leiman J. M. (1957). The development of hierarchical factor solutions. Psychometrika 22, 53–61. doi: 10.1007/BF02289209

[ref54] Schooler J. W. Smallwood J. Christoff K. Handy T. C. Reichle E. D. Sayette M. A. (2011). Meta-awareness, perceptual decoupling and the wandering mind. Trends Cogn. Sci. 15, 319–326. doi: 10.1016/j.tics.2011.05.006, PMID: 21684189

[ref55] Seli P. Carriere J. S. A. Smilek D. (2015). Not all mind wandering is created equal: dissociating deliberate from spontaneous mind wandering. Psychol. Res. 79, 750–758. doi: 10.1007/s00426-014-0617-x, PMID: 25284016

[ref56] Smallwood J. Davies J. B. Heim D. Finnigan F. Sudberry M. O’Connor R.. (2004). Subjective experience and the Attentional lapse: task engagement and disengagement during sustained attention. Conscious. Cogn. 13, 657–690. doi: 10.1016/j.concog.2004.06.003, PMID: 15522626

[ref57] Smallwood J. O’Connor R. C. (2011). Imprisoned by the past: unhappy moods lead to a retrospective bias to mind wandering. Cognit. Emot. 25, 1481–1490. doi: 10.1080/02699931.2010.545263, PMID: 21432633

[ref58] Smallwood J. Schooler J. W. (2006). The restless mind. Psychol. Bull. 132, 946–958. doi: 10.1037/0033-2909.132.6.946 17073528

[ref59] Smallwood J. Schooler J. W. (2015). The science of mind wandering: empirically navigating the stream of consciousness. Annu. Rev. Psychol. 66, 487–518. doi: 10.1146/annurev-psych-010814-015331, PMID: 25293689

[ref60] Spitzer R. L. Kroenke K. Williams J. B. W. Löwe B. (2006). A brief measure for assessing generalized anxiety disorder: the GAD-7. Arch. Intern. Med. 166, 1092–1097. doi: 10.1001/archinte.166.10.1092 16717171

[ref61] Stawarczyk D. Majerus S. Catale C. D’Argembeau A. (2014). Relationships between mind-wandering and attentional control abilities in young adults and adolescents. Acta Psychol. 148, 25–36. doi: 10.1016/j.actpsy.2014.01.007, PMID: 24486804

[ref62] Stawarczyk D. Majerus S. D’Argembeau A. (2013). Concern-induced negative affect is associated with the occurrence and content of mind-wandering. Conscious. Cogn. 22, 442–448. doi: 10.1016/j.concog.2013.01.012, PMID: 23466878

[ref63] Tabachnick B.G. Fidell L.S. (2001). Using multivariate statistics. 4th Edition, Allyn and Bacon. Boston, MA.

[ref64] Tortella-Feliu M. Morillas-Romero A. Balle M. Bornas X. Llabrés J. Pacheco-Unguetti A. P. (2014). Attentional control, attentional network functioning, and emotion regulation styles. Cognit. Emot. 28, 769–780. doi: 10.1080/02699931.2013.860889 24295123

[ref65] Treynor W. Gonzalez R. Nolen-Hoeksema S. (2003). Rumination reconsidered: a psychometric analysis. Cogn. Ther. Res. 27, 247–259. doi: 10.1023/A:1023910315561

[ref900] Unsworth Nash. Brittany D McMillan. Gene A Brewer. Gregory J Spillers. (2012). “Everyday attention failures: an individual differences investigation”. J. Exp Psychol Learn Mem Cogn. 38, 1765–75. doi: 10.1037/a0028075 22468805

[ref66] Watson D. Clark L. A. Tellegen A. (1988). Development and validation of brief measures of positive and negative affect: the PANAS scales. J. Pers. Soc. Psychol. 54, 1063–1070. doi: 10.1037/0022-3514.54.6.1063, PMID: 3397865

[ref67] Yamaoka A. Yukawa S. (2020). Does mind wandering during the thought incubation period improve creativity and worsen mood? Psychol. Rep. 123, 1785–1800. doi: 10.1177/0033294119896039, PMID: 31856642

